# Cucumber *SUPERMAN* Has Conserved Function in Stamen and Fruit Development and a Distinct Role in Floral Patterning

**DOI:** 10.1371/journal.pone.0086192

**Published:** 2014-01-23

**Authors:** Jianyu Zhao, Meiling Liu, Li Jiang, Lian Ding, Shuang Shuang Yan, Juan Zhang, Zhaobin Dong, Huazhong Ren, Xiaolan Zhang

**Affiliations:** 1 Department of Vegetable Sciences, Beijing Key Laboratory of Growth and Developmental Regulation for Protected Vegetable Crops, China Agricultural University, Beijing, P. R. China; 2 National Maize Improvement Center of China, Key Laboratory of Crop Genetic Improvement and Genome of Ministry of Agriculture, Beijing Key Laboratory of Crop Genetic Improvement, China Agricultural University, Beijing, P. R. China; National Taiwan University, Taiwan

## Abstract

The *Arabidopsis SUPERMAN (SUP)* gene encodes a C2H2 type zinc finger protein that is required for maintaining the boundaries between stamens and carpels, and for regulating development of ovule outer integument. Orthologs of *SUP* have been characterized in bisexual flowers as well as dioecious species, but it remains elusive in monoecious plants with unisexual flowers on the same individual. Here we isolate the *SUP* ortholog in *Cucumis sativus L* (*CsSUP*), a monoecious vegetable. *CsSUP* is predominantly expressed in female specific organs: the female flower buds and ovules. Ectopic expression of *CsSUP* in *Arabidopsis* can partially complement the fruit development in *sup-5* mutant, and its over-expression in wide-type leads to reduced silique length, suppressed stamen development and distorted petal patterning. Our data suggest that *CsSUP* plays conserved as well as distinct roles during flower and fruit development, and it may function in the boundaries and ovules to balance petal patterning, stamen and ovule development in *Arabidopsis*.

## Introduction

In flowering plants, approximately 90% species produce bisexual flowers with specialized male and female organs in the same flower, while 6% species are dioecious with separate male and female plants, and the remaining species are monoecious, producing male and female flowers on the same individual [1], [2]. *Arabidopsis thaliana* is a model species for bisexual plant, whose flowers consist of four types of floral organs arranged into concentric whorls, specifically sepals in outermost (whorl 1), petals in whorl 2, stamens in whorl 3 and carpels in innermost (whorl 4). The patterning of floral organs in different whorls is controlled by the combinatorial interactions between three classes of homeotic genes, designated A, B, and C class of genes [3], [4]. Sepal identity is determined by the function of A genes *APETALA1* and *APETALA2*, petal identity is specified by the simultaneous function of A and B genes (which include *APETALA3* and *PISTILLATA*), stamen identity is determined by combinatorial action of B and C (provided by the *AGAMOUS* gene), whereas carpel identity is specified by C function alone [3]–[5]. Recently, D class of genes, *FLORAL BINDING PROTEIN7 (FBP7)* and *FLORAL BINDING PROTEIN11 (FBP11)*, have been shown to be involved in carpel and ovule development, whereas E class of genes (provided by *SEPALLATA 1-4*) are required for all the floral organ development, thus the classical ABC model has been modified into ABCDE model for floral patterning [6]–[10].

In Arabidopsis, the cadastral gene *SUPERMAN (SUP)* has shown to be a negative regulator of B class of genes at the boundaries between stamens and carpels. Loss of function of *AtSUP* leads to extra stamen production in the fourth whorl at the expense of carpel development, and expansion of the B class of genes *APETALA3* and *PISTILLATA* from the second and third whorls into the forth whorl [11], [12]. *AtSUP* encodes a C2H2 type zinc finger transcription factor that is expressed in the subdomain of third whorl adjacent to the forth whorl, and may function at the boundaries to balance cell proliferation in whorl 3 and 4 [12], [13]. The DLELRL hexapeptide in the AtSUP carboxy terminal domain confers the transcriptional repression activity and is required for the normal flower development [14], [15]. In addition, *AtSUP* also function in cell proliferation suppression of outer integument on the adaxial side of the ovule [16]. In *sup* mutants, outer integument grows evenly and leads to production of infertile ovules that are radially symmetrical [16]. Consistent with the function in ovule, *AtSUP* is expressed in the developing ovules and then limited to the stalks of ovules [16].

Numerous studies have explored the function of *AtSUP* upon ectopic expression in various species. Overexpression of *AtSUP* under *APETALA1* promoter leads to reduced size as well as decreased number of all four types of floral organs in *Arabidopsis* [17]. Similarly, ectopic expression of *AtSUP* using *FLORAL BINDING PROTEIN1 (FBP1)* promoter results in size reduction in petals and stamens in petunia or tobacco [18]. However, when overexpressed under constitutive promoter in dicotyledonous species such as *Arabidopsis* or tobacco, *AtSUP* causes dwarfness at the whole plant level, and restores stamen development and produces functional pollen in an alloplasmic CMS tobacco plant [14], [19], [20]. While overexpressed in monocotyledon such as rice, high level of *AtSUP* expression leads to juvenile death, and low level of *AtSUP* expression results in expansion of ventral carpel and decreased number of stamen [21]. Recently, several orthologs of *SUP* have been cloned, including *NtSUP* in *Nicotiana tabacum*, *PhSUP1* in *Petunia hybrida* and *SlSU*P in *Silene latifolia*, and their functions are generally conserved, but the expression patterns are quite divergent [19], [20], [22].

Despite the extensive studies of *AtSUP* and *SUP* orthologs, no study has been performed in monoecious plant with unisexual flowers on the same individual. Cucumber (*Cucumis sativus L.*) is a typical monoecious species with male flowers produce at the bottom and female flowers form at the top. Therefore, we cloned the *SUP* ortholog in cucumber designated as *CsSUP*, and the expression pattern of *CsSUP* was characterized by both qRT-PCR and *in situ* hybridization. *CsSUP* is predominantly expressed in female organs: female flower buds and ovules of fruit. Ectopic expression of *CsSUP* in *Arabidopsis* can partially complement the fruit development in *sup-5* mutant, and its over-expression in wide-type leads to reduced silique length, suppressed stamen development, and disorganized petal patterning. Our data suggested that *CsSUP* possesses conserved functions in stamen and fruit development as well as a distinct role in floral patterning.

## Results

### Identification of *CsSUP* gene from *C. sativus L*

**Figure 1:**
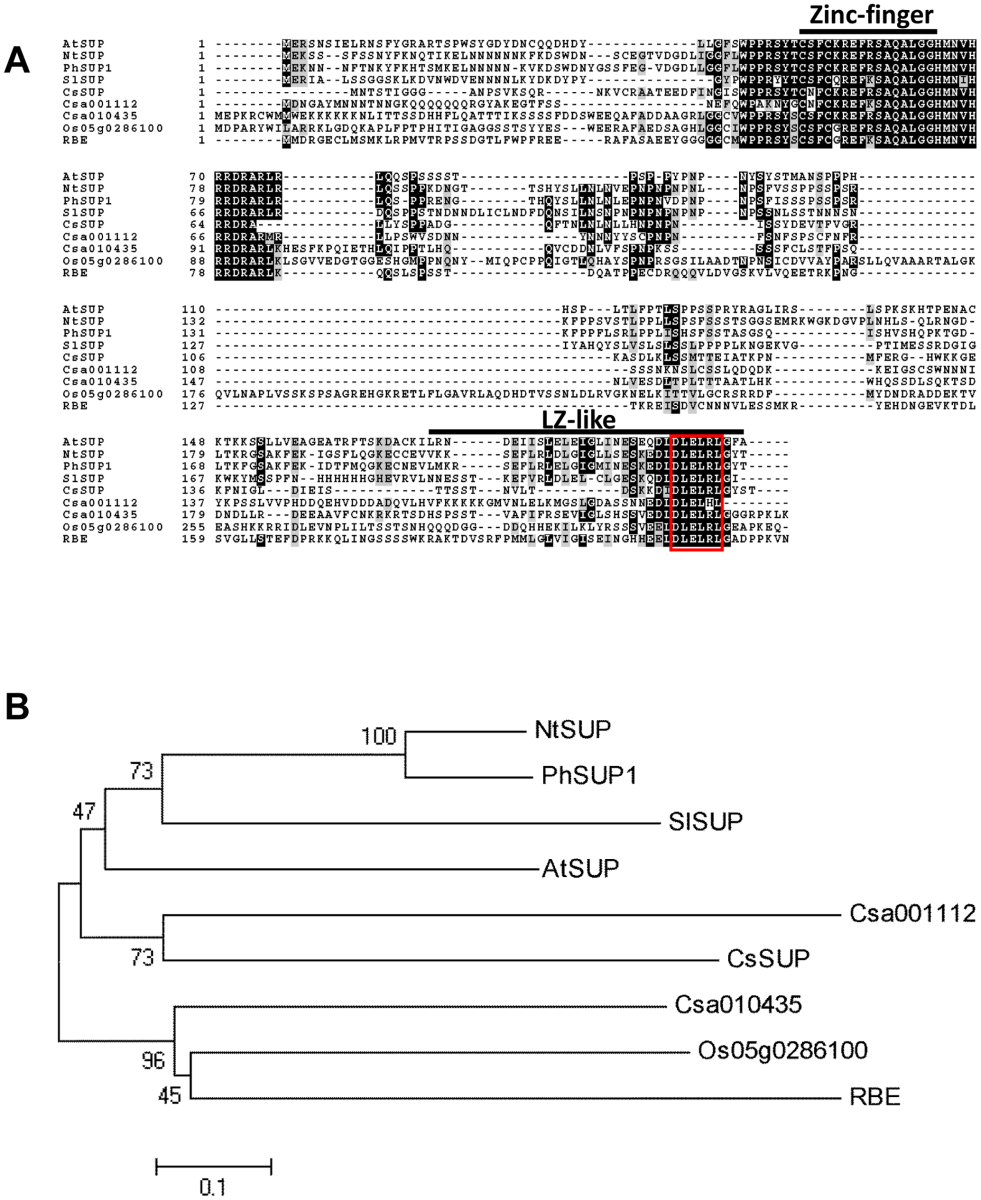
Alignment and phylogenetic analysis of *SUP*-like genes. (**A**) Alignment of *CsSUP* and *SUP*-like genes. The amino acid sequences of CsSUP, Csa001112 and Cas010435 in cucumber, AtSUP and RBE in *Arabidopsis*, NtSUP in tobacco, PhSUP in petunia, SlSUP in white campion and Os05g0286100 in rice were aligned using ClustalW in the MEGA5 software package. The black and gray areas indicate identical and similar amino acid, respectively. Zinc-finger and leucine zipper (LZ)-like domains were indicated in black lines. The DLELRL domain [15] was showed in red box. (**B**) An unrooted phylogenetic tree constructed using the amino acid sequences of CsSUP, Csa001112, Cas010435, AtSUP, RBE, NtSUP, PhSUP, SlSUP and Os05g0286100 based on the neighbor joining method. Branch length is proportional to evolutionary distance.

To isolate the potential *SUP-like* genes in cucumber, we performed BLAST analysis against Cucumber Genome Database [23] using the sequence information of *AtSUP* in *Arabidopsis*. Three *SUP-like* genes were identified in cucumber, *Csa000134*, *Csa001112* and *Cas010435*, in which *Csa000134* displays the lowest e-value with *AtSUP* using both cDNA (3e-16) and deduced protein sequence (2e-17), thus was designated as *CsSUP* in this study. The cDNA of *CsSUP* were cloned from the female buds. Consistent with known *SUP* orthologs [12], [20], [22], [24], *CsSUP* has no intron, and the full length *CsSUP* encodes a protein of 171 amino acids. ClustalW was used to align the amino acid sequence of CsSUP with known *SUP-like* genes, including AtSUP and RABBIT EARS (RBE) in *Arabidopsis*, NtSUP in tobacco, PhSUP in petunia, SlSUP in white campion, and Os05g0286100 in rice [25] (Figure 1A). Despite CsSUP shows only 28.78%, 33.62%, 29.78% and 30.36% identity with AtSUP,NtSUP,PhSUP and SlSUP respectively, the zinc-finger domains and the leucine zipper (LZ)-like domains are highly conserved (Figure 1A). The DLELRL domains, which are required for transcriptional repression during cell proliferation in *Arabidopsis* and petunia [14], [15], are identical over all the proteins we aligned except for *Csa001112*. Phylogenetic analysis was performed with the entire amino acid sequences using the neighbor-joining (NJ) method [26]. As shown in Figure 1B, *CsSUP* and *Csa001112* belong to the same clade with known SUP orthologs, which are distinct from the clade consisting of *SUP-like* genes *RBE*, *Os05g0286100* and *Cas010435* [25], suggesting that *CsSUP* and *Csa001112* maybe the *SUP* homologues in cucumber.

### Expression pattern of *CsSUP* in Cucumber

**Figure 2:**
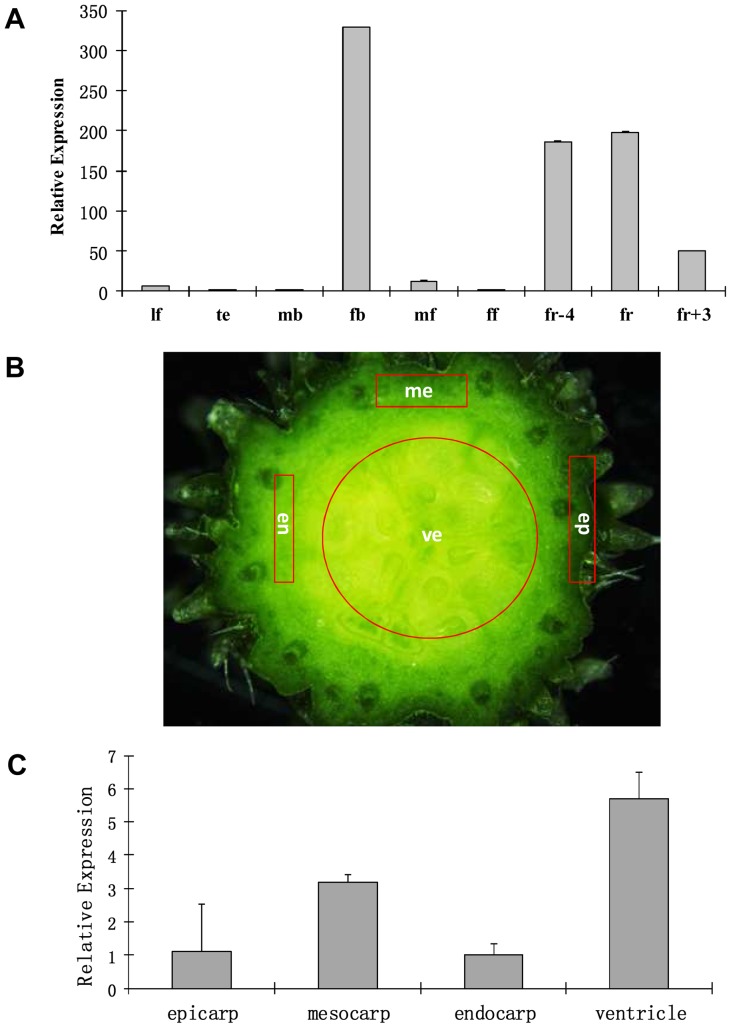
Quantitative RT-PCR (qRT-PCR) analysis of *CsSUP* in different organs of cucumber (A) or different parts of cucumber fruit (B–C). Three biological replicates were used for each sample, and 18S rRNA was used as internal control. Bars represent the standard error. (**A**) *CsSUP* is predominately expressed in the female flower bud and developing fruit. lf: leaves, te: tendrils, mb: male flower buds, fb: female flower buds, mf: male flowers, ff: female flowers, fr-4: fruit of 4 days before flower opening, fr: fruit on flower opening, fr+3: fruit of 3 days after flower opening. (**B**) Transverse sections of commercially mature cucumber fruit. ep: epicarp, me: mesocarp, en:endocarp, ve:ventricle. (**C**) *CsSUP* is highly expressed in the ventricle of cucumber fruit where the ovules are located.

Next, we examined the transcription of *CsSUP* and *Csa001112* in different organs of cucumber through qRT-PCR (Figure 2A). Total RNA was isolated from leaves (lf), tendril (te), male flower buds (mb), female flower buds (fb), male open flowers (mf), female open flowers (ff), and fruits at three different developmental stages. Our results showed that *CsSUP* and *Csa001112* display similar expression patterns, which are highly enriched in female specific tissues such as female flower buds and developing fruits, whereas there were very few expressions in other organs (Figure 2A, Figure S1). Specifically, *CsSUP* has the highest expression in female flower buds (Figure 2A), while *Csa001112* has the most abundant transcription in fruits 4 days before flower opening (fr-4) (Figure S1). Given that *CsSUP* shows the highest sequence similarity with *SUP* orthologs, and the expression of *CsSUP* appears to be enriched in younger female tissues than that of *Csa001112*, we focus on characterization of *CsSUP* thereafter. Cucumber fruit largely results from expansion of ovary, therefore, we next explored the transcript accumulation of *CsSUP* in different parts of cucumber fruits (Figure 2B–C). Epicarp, mesocarp, endocarp and ventricle were separated according to Figure 2B, and qRT-PCR was performed with these four types of tissues. Ventricle, where the ovules are located, showed the highest *CsSUP* expression (over 5 folds than that of epicarp), and mesocarp, which comprised of plentiful vascular system, displayed the second highest expression among the four parts of fruit (Figure 2C), suggesting that *CsSUP* may be highly expressed in ovules and vasculature of cucumber fruits.

**Figure 3:**
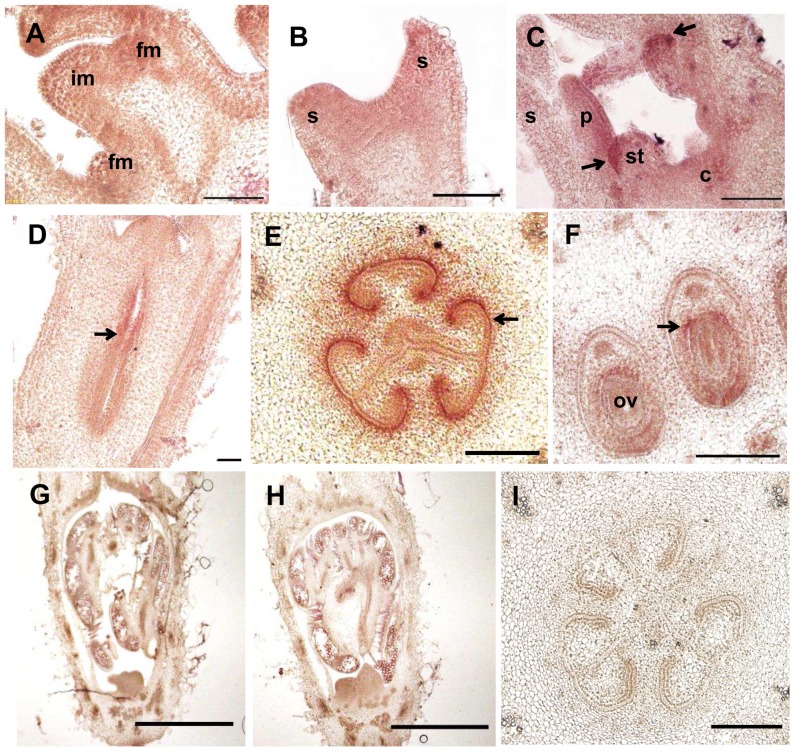
*In situ* hybridization of *CsSUP* transcripts in developing flowers and fruits of cucumber. Longitudinal sections of the shoot apex (A), stage 2 flower (B), stage 4 flower (C) and stage 8 female flower (D) reveal that *CsSUP* is expressed throughout in the IM, FM and young floral primordia (stage 1–2), and then limited to the boundary between petal and stamen (arrow in C), and the developing ovary (arrow in D). Transverse sections reveal that *CsSUP* is specifically expressed in the boundary of developing ovary (arrow in E), and the developing ovules (arrow in F). *CsSUP* is undetectable in the male flower primordia (G). Control hybridizations with *CsSUP* sense probe in male (H) or female flower primordia (I) show no signal. Bar = 200 µm except for (I), in which bar = 100 µm.

We further characterized the spatial and temporal expression pattern of *CsSUP* during flower and fruit development through *in situ* hybridization (Figure 3). Consistent with the results in qRT-PCR, *CsSUP* expression is detected throughout the inflorescence meristem (IM), floral meristem (FM) and young floral primordia during stage 1–2 [27]. By flowers develop into stage 4, *CsSUP* expression is limited to the boundary between petal and stamen (arrow in Figure 3C). *CsSUP* shows high expression in the developing ovary of female flower (arrow in Figure 3D), specifically, in the boundary of developing ovary (arrow in Figure 3E), and the developing ovules (arrow in Figure 3F). In male flower primordia (Figure 3G), *CsSUP* shows no detectable signal as compared to the sense control hybridizations (Figure 3H–I).

### Phenotypes of ectopic *CsSUP* expression in *Arabidopsis*

**Figure 4:**
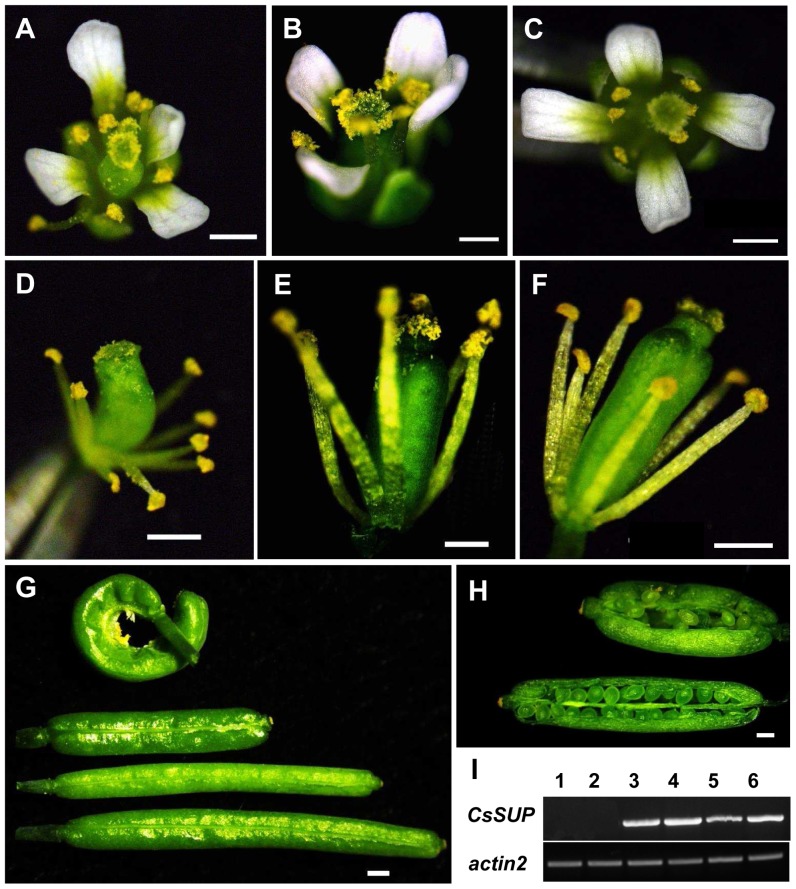
Ectopic *CsSUP* expression can partially rescue the phenotype of *sup-5* mutant *Arabidopsis*. Flowers of *sup-5* (A, D), *pAtSUP::CsSUP;sup-5* (B, E) and *35S::CsSUP;sup-5* (C, F) shows the complement of excess stamen upon ectopic *CsSUP* expression. (G) Representative siliques of *sup-5* (top), *pAtSUP::CsSUP;sup-5* (top middle), *35S::CsSUP;sup-5* (bottom middle) and *Ler* (bottom) indicate the partial rescue of the *sup-5* silique development by ectopic expression of *CsSUP in Arabidopsis*. (H) Opened siliques of *sup-5 (top)* and *pAtSUP::CsSUP;sup-5* (bottom) at similar developmental stages. (I) Expression of *CsSUP* in transgenic *Arabidopsis*. Lane 1–2: *sup-5* plants, lane 3–4: *35S::CsSUP;sup-5* lines, lane 5–6: *pAtSUP::CsSUP;sup-5* lines. *Actin2* was used as internal control to normalize the expression data. Bars = 1 mm.

To understand the function of *CsSUP*, ectopic expression analysis was first performed in the *sup-5* mutant *Arabidopsis* plants under *AtSUP* promoter (*pAtSUP*) or 35S promoter of *Cauliflower mosaic virus* (CaMV). As shown in Figure 4, ectopic *CsSUP* expression can partially rescue the *sup-5* mutant phenotype. In the *sup-5* mutant, number of stamens and carpels are increased, silique length is decreased and seed number is reduced (Figure 4A, 4D, 4G and 4H) [28], ectopic expression of *CsSUP* under native *AtSUP* promoter *(pAtSUP::CsSUP;sup-5)* (Figure 4B, 4E,4G and 4H) or constitutive 35S promoter *(35S::CsSUP;sup-5)* (Figure 4C, 4F and 4G) results in normal number of stamens and partially rescued silique length and morphology. For example, the ratio of normal silique is 23% in the *sup-5* mutant, and it increases to 50% in the *pAtSUP::CsSUP;sup-5*, and to 83% in the *35S::CsSUP;sup-5* (Table 1). Further, *35S::CsSUP;sup-5* generally has better rescue effects than that of *pAtSUP::CsSUP;sup-5* with regards to both flower and silique development (Figure 4). Consistently, expression of *CsSUP* is higher in the *35S::CsSUP;sup-5* lines as compared to those in the *pAtSUP::CsSUP;sup-5* lines (Figure 4I). We further quantified the seed numbers in *sup-5* and *35S::CsSUP;sup-5*. As shown in Table 2, ectopic expression of *CsSUP* significantly increased the seed numbers per silique in the *sup-5* mutant background, suggesting that the function of SUP is largely conserved between complete flower *Arabidopsis* and unisexual flower cucumber.

**Table 1:** Quantification of silique phenotype.

	Normal	Mild malformed	Severe malformed	Normal %
*sup-5*	7	5	18	23.33%
*pAtSUP::CsSUP;sup-5*	15	4	11	50.00%
*35S::CsSUP;sup-5*	25	2	3	83.33%

The values shown are silique numbers. Total of 30 siliques were characterized for each genotype.

**Table 2:** Partial complement of *sup-5* mutant upon ectopic expression of *CsSUP*.

Control Plant	Seed Number	Transgenic Plants		Seed Number
*Ler*	46.10±0.78	*35S::CsSUP/sup-5*	line1	23.67±1.20**
*sup-5*	14.80±0.64		line2	26.00±0.87**
			line3	21.33±1.61**
			line4	30.67±1.98**
			Average	25.41±4.81**

The values shown are the means ± SE of 20 siliques from *Arabidopsis Ler* and *sup-5* mutant, or 6 siliques from *CsSUP* transgenic lines (*35S::CsSUP/sup-5*). T-tests were used to determine whether differences between *sup-5* and transgenic lines were statistically significant.

* and ** represent p<0.05 and p<0.01, respectively.

**Figure 5:**
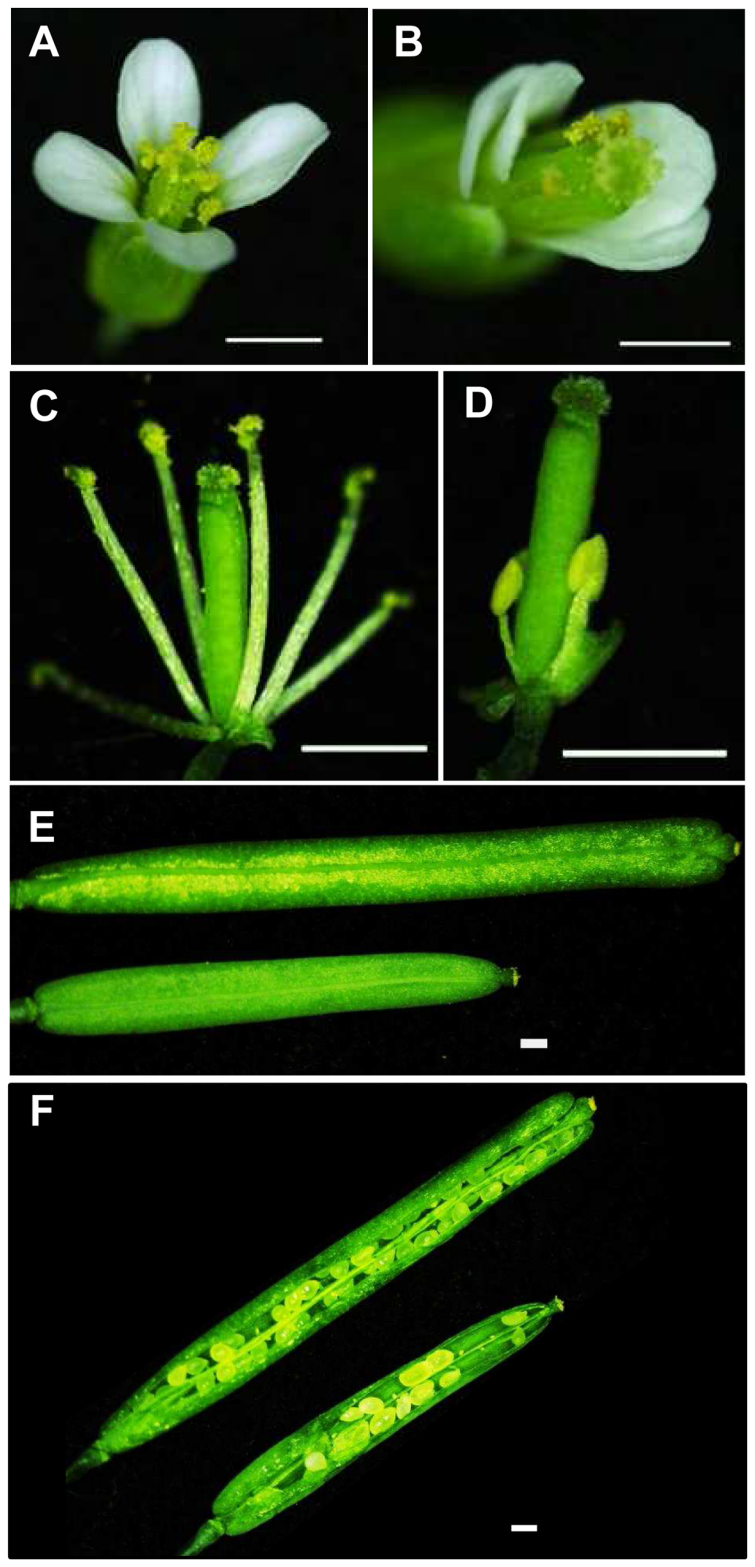
Phenotypes of overexpression of *CsSUP* in wild type *Arabidopsis*. (A–B) Flowers of wild type (A) and *35S::CsSUP* (B) show the disturbed petal organization. (C–D) Stamens of wild type (C) and *35S::CsSUP* (D) indicate the suppressed stamen development in the transgenic lines. (E–F) Siliques (E) and opened siliques (F) of wild type (top) and *35S::CsSUP* (bottom) show the reduced silique length and decreased seed numbers. Bars = 1 mm.

Next, we examined the effects upon overexpression of *CsSUP* in the *Ler* background (*35S::CsSUP/Ler*). As indicated in Figure 5, overexpression of *CsSUP* leads to disorganized petal patterning, suppressed stamen development, and reduced silique length (Figure 5). Compared to the flowers in wide-type plant (Figure 5A), *35S::CsSUP/Ler* transgenic flowers display 10–20% petals with aberrant organization (Figure 5B), while petal number and petal size show no noticeable changes. Further, stamen development in the *35S::CsSUP/Ler* transgenic flowers is largely suppressed. In contrast to the wild-type flower with six stamens, the *35S::CsSUP* transgenic flowers developed an average of 4.39±0.06 stamens (Table 3), and about 10–15% stamens are greatly short (Figure 5C–D). Accordingly, silique length and seed numbers per silique are significantly reduced in the *35S::CsSUP* plants (Figure 4E–F, Table 4 and Table 5). For example, the average silique length is 10 mm with 46 seeds per silique in the WT, while it decreases to 6.8 mm with 17 seeds per silique in the *35S::CsSUP* transgenic plants (Table 4 and Table 5). At the whole plant level, transgenic plants are slightly dwarf as compared to wide-type plant (data not shown). Taken together with the expression data of *CsSUP* in Figure 2 and 3, *CsSUP* may function in the boundaries and ovules to modulate petal patterning, stamen and fruit development in *Arabidopsis*, probably through negative regulation of cell proliferation.

**Table 3:** Reduced stamen numbers in *35S::CsSUP* transgenic plants.

Control Plant	Stamen Number	Transgenic Plants		Stamen number
*Ler*	6.0±0.0	*35S::CsSUP/Ler*	line1	3.97±0.13**
			line2	4.44±0.11**
			line3	4.38±0.13**
			line4	4.76±0.08**
			Average	4.39±0.06**

The values shown are the means ± SE of 30 flowers from wild-type *Arabidopsis*, or 20 flowers from *CsSUP* transgenic plants (*35S::CsSUP/Ler*). T-tests were used to determine whether differences between *Ler* and transgenic lines were statistically significant.

* and ** indicate p<0.05 and p<0.01, respectively.

**Table 4:** Reduced silique length in *35S::CsSUP* transgenic plants.

Control Plant	Silique Length (mm)	Transgenic Plants		Silique Length (mm)
*Ler*	10.19±0.14	*35S::CsSUP*/Ler	line1	6.90±0.10**
			line2	5.08±0.10**
			line3	8.53±0.14**
			Average	6.84±0.16**

The values shown are the means ± SE of 30 siliques from *Arabidopsis Ler* or *CsSUP* transgenic lines (*35S::CsSUP/Ler*). T-tests were used to determine whether differences between *Ler* and transgenic lines were statistically significant.

* and ** represent p<0.05 and p<0.01, respectively.

**Table 5:** Reduced seed numbers in *35S::CsSUP* transgenic plants.

Control Plant	Seed number/Silique	Transgenic Plants		Seed number/Silique
*Ler*	46.10±0.78	*35S::CsSUP/Ler*	line1	17.35±0.97**
			line2	7.15±0.49**
			line3	26.85±1.56**
			Average	17.12±1.22**

The values shown are the means ± SE of 20 siliques from *Arabidopsis Ler* or *CsSUP* transgenic lines (*35S::CsSUP/Ler*). T-tests were used to determine whether differences between *Ler* and transgenic lines were statistically significant.

* and ** show p<0.05 and p<0.01, respectively.

## Discussion

During early stages of flower development, all floral buds are bisexual, and unisexual flowers are formed by subsequent arrestment of either carpel or stamen development [29]. Phylogenetic analysis indicated that unisexual flowers evolved from bisexual flowers many times in the angiosperm lineage [20], [30], [31]. It is not surprising that unisexual species and bisexual species have distinct mechanism underlining flower development. So far, most studies about floral patterning are performed in bisexual flowers, while flower development in unisexual species is largely neglected. Here we cloned the ortholog of *AtSUP* in unisexual species cucumber (*CsSUP*) (Figure 1), and we characterized the expression pattern of *CsSUP* by tissue-specific qRT-PCR and *in situ* hybridization (Figures 2 and 3), and we explored the function of *CsSUP* by ectopic expression in *Arabidopsis* (Figures 4 and 5, Tables 1–5). Our data showed that *CsSUP* played shared as well as divergent roles during flower and fruit development.

### *CsSUP* is expressed mostly in the female specific organs

Among the orthologs of *SUP*, expression patterns of *PhSUP* and *SlSUP* have been explored previously [13], [20], [22]. Transcripts of *Arabidopsis SUP* were first detected in late stage 3 flower primordia, and in boundaries between stamen and carpel primordia. Then, it was found in the adaxial side of the stamen primordia and later in stage 9, *SUP* RNA was detected in the ovary [13]. *PhSUP*, on the other hand, is expressed in the basal regions of developing petals and stamens, the interthecal regions of developing anthers, and the basal part of ovules [20]. While the *SlSUP* gene is exclusively expressed in female flowers in *Silene latifolia*, in developing petals, stamens and ovules [22]. In this study, we found that *CsSUP* is mostly expressed in the female specific organs: the female flower buds and fruits (Figure 2). Specifically, *CsSUP* is expressed throughout the IM, FM, and stage 1–2 floral primordia, then it is restricted to the boundary between petal and stamen at stage 4. By the time of unisexual flower is noticeable (stage 6 and on), *CsSUP* is expressed only in female flowers, in the boundary of developing ovary and in ovules (Figure 3). Therefore, the expression patterns of SUP orthologs are quite divergent, implying they may have distinct roles in bisexual flowers, dioecious species or monoecious species. Despite the specific domains are different, both *CsSUP* and *SlSUP* are expressed predominantly in female flowers, the reason of which may lie in the fact that both species produce unisexual flowers.

### *CsSUP* has both conserved and divergent functions during flower and fruit development

*Arabidopsis AtSUP* has been shown to function through cell proliferation to regulate the balance between stamen fate and carpel fate, as well as to control the growth of outer integument of ovule [13], [16]. Loss-of-function of *AtSUP* results in increased number of stamen, defective carpel and infertile ovules [13], [16]. Ectopic expression of *PhSUP* or *SlSUP* in *Arabidopsis* can partially or fully rescue the excess stamen and infertile ovule phenotype in *sup* mutants [20], [22]. Similarly, our study showed that overexpression of *CsSUP* can partially complement the *sup-5* mutant phenotype, particularly the number of stamens, silique length and number of seeds per silique (Figure 4, Tables 1–2), suggesting that *CsSUP* plays a conserved role during stamen and ovule development. However, constitutive expression of *CsSUP* in wild type *Arabidopsis* leads to disorganized patterning of petal, reduced number of stamen, decreased length of silique, reduced seed production and mild dwarfness of the whole plant (Figure 4). Dwarfness, suppressed petal and stamen development have been reported previously when ectopic expression of *AtSUP* or *SUP* orthologs under the 35S promoter [14], [19], [20], [22], while decreased silique length and seed production, and disorganized petal patterning appears to be specific to *CsSUP*, suggesting that monoecious *CsSUP* may evolved a distinct roles in petal patterning and fruit development as compared to *SUP* orthologs in bisexual or dioecious species. Considering the unique expression of *CsSUP* in the boundary between petal and stamen primordia (Figure 3C), the boundary of developing ovary (Figure 3E), and in ovules (Figure 3F), *CsSUP* may function in the boundaries and ovules to modulate petal patterning, stamen and fruit development in *Arabidopsis*, probably through negative regulation of cell proliferation. Previous studies showed that depending on the species, cell types and expression level, *AtSUP* and *SUP* orthologs stimulated or suppressed cell division, cell expansion or cell elongation so that enabling proper flower and ovule development [17], [18], [20], [21], [24], [32]. Recent studies showed that *Arabidopsis* and tobacco *SUPs* regulated cell proliferation through auxin and cytokinin signaling pathways in tobacco [24], and *AtSUP* suppresses cell division in floral meristem redundantly with a RNA helicase CARPEL FACTORY [33], and epigenetic modification-methylation is involved in the active or inactive state of *AtSUP* [34], [35] Therefore, tackling the underlining mechanism of *CsSUP* function in *Arabidopsis*, especially the potential relationship between *CsSUP* with hormones, RNA helicase and methylation modification during petal patterning and silique development, would be useful for gaining an entrance into understand how *CsSUP* might function in cucumber. Further studies through RNA interference and genetic transformation, to explore the precise roles of *CsSUP* in cucumber would shed light on the evolutional scenery of SUP function in hermaphrodite, dioecious and monoecious species of flowering plants.

## Materials and Methods

### Plant materials and growth

Cucumber (*Cucumis sativus L.*) inbred line 1461 was used in this study. Seeds were grown in pots in a regulated chamber at 28°C in a 16 h light/8 h dark cycle for about a month, and then they were moved to greenhouse for further development. The *Arabidopsis Landsberg erecta* (*Ler*) was used as wide-type control in the transgenic analysis. The *sup-5* mutant was obtained from the Arabidopsis Biological Resource Center (ABRC). *Arabidopsis* seeds were sown in soil or Murashige-Skoog (MS) medium (0.2% agar and 1% sucrose) at 23°C on a 16 h light/8 h dark cycle.

### Isolation of *CsSUP*

Total RNA was extracted from female flower buds using the Huayueyang RNA isolation kit (China), and 3 µg of samples were used to synthetize cDNA using M-MLV Reverse Transcriptase (Promega). The cDNA samples were amplified by PCR via the following system: 95°C for 5 min; 30 cycles of 95°C for 30 s, 53°C for 30 s, and 72°C for 30 s; then 72°C for 10 min.

### Sequence comparison and phylogenetic analysis

Amino acid sequences of CsSUP and SUP-like genes were obtained by BLAST searches (http://www.ncbi.nlm.nih.gov/BLAST/) and aligned by ClustalW software (http://clustalw.ddbj.nig.ac.jp/top-j.html/). A phylogenetic tree based on their entire amino acid sequences was constructed by applying the neighbor joining (NJ) method using the bootstrap analysis with1000 replications [26]. The GeneBank accession numbers of the amino acid sequences are: AtSUP (U38946), PhSUP1 (AB117749), NtSUP (GQ227844), SlSUP (BAH59432), RBE (AB107371), Os05g0286100 (BAF17009). The sequence data for the three *SUP-like* genes in cucumber can be found in the Cucumber Genome Initiative databases (http://cucumber.genomics.org.cn) under the following accession numbers: CsSUP (Csa000134), Csa001112 and Cas010435.

### Quantitative real-time RT-PCR

The leaves, tendrils, flower buds, flowers and fruits were frozen in liquid nitrogen and stored at −80°C prior to RNA extraction. Total RNA was extracted with Huayueyang RNA isolation kit (China), cDNA was synthetized using M-MLV Reverse Transcriptase (Promega). Quantitative RT-PCR was performed using an Applied Biosystems 7500 real-time PCR systems with SYBR Green as fluorescent dyes (TaKaRa). Three biological replicates were performed, upon which three technical replicates were used for the qRT-PCR analysis. 18S rRNA was used as reference control to normalize the expression data [36]. The gene specific primers are listed in Table S1.

### Semi-quantitative RT-PCR

Inflorescence of *sup-5*, *pAtSUP::CsSUP;sup-5, 35S::CsSUP;sup-5* were frozen in liquid nitrogen and stored at −80°C prior to RNA extraction. Total RNA was extracted with Huayueyang RNA isolation kit (China), cDNA was synthetized using M-MLV Reverse Transcriptase (Promega). *Actin2* was used as internal control to normalize the expression data. The gene specific primers are listed in Table S1.

### *In situ* hybridization

Cucumber flower buds and young fruits were fixed, embedded, sectioned, and hybridized with digoxigenin (DIG)-labeled sense and antisense RNA probes as described [37]. Through PCR amplification of cDNA, *in situ* probes were synthesized using gene specific primers including T7 and SP6 RNA polymerase-binding sites. T7 RNA polymerase was used for the synthesis of antisense probes and SP6 RNA polymerase was used for the generation of sense probes. The primer pairs are listed in Table S1.

### Ectopic expression of *CsSUP* in *Arabidopsis*

The full-length *CsSUP* cDNA fragment was amplified by PCR using gene specific primers *O-CsSUP-F* and *O-CsSUP-R* containing Xba I and Sma I restriction enzyme sites respectively. The resulting fragment was digested and fused into the pBI121 vector. The resulting *CsSUP*-pBI121 construct driven by CaMV35S promoter was introduced to *Agrobacterium tumefaciens* by electric shock to transform wide-type (*Ler*) and *sup-5* mutant of *Arabidopsis* using the floral-dip method [38]. Meantime, The 2.2 kb length of *AtSUP* promoter was amplified by PCR using gene specific primers *pAtSUP-F* and *pAtSUP-F* containing Cla I and Xba I restriction enzyme sites respectively. The resulting fragment was digested and fused into the *CsSUP*-pBI121 construct, and transformed into *sup-5* mutant of as above. Transgenic plants were screened by MS medium with 40 mg/L kanamycin, and resistant plants were verified by *CsSUP* specific primers *CsSUP-F* and *CsSUP-R*.

## Supporting Information

Figure S1 **Quantitative RT-PCR (qRT-PCR) analysis of** ***Cs001112*** **in different organs of cucumber.** *Csa001112* is predominately expressed in the fruit. lf: leaves, te: tendrils, mb: male flower buds, fb: female flower buds, mf: male flowers, ff: female flowers, fr-4: fruit of 4 days before flower opening, fr: fruit on flower opening, fr+3: fruit of 3 days after flower opening. Three biological replicates were used for each sample, and 18S rRNA was used as internal control. Bars represent the standard error.
(TIF)

Table S1 **Oligonucleotide primers used in this study.**
(DOCX)
