# Supplementary figures and images for: Cucumber SUPERMAN Has Conserved Function in Stamen and Fruit Development and a Distinct Role in Floral Patterning
Source: PLoS One. 2014 Jan 23;9(1):e86192. doi: 10.1371/journal.pone.0086192 (PMC3900519; doi:10.1371/journal.pone.0086192)

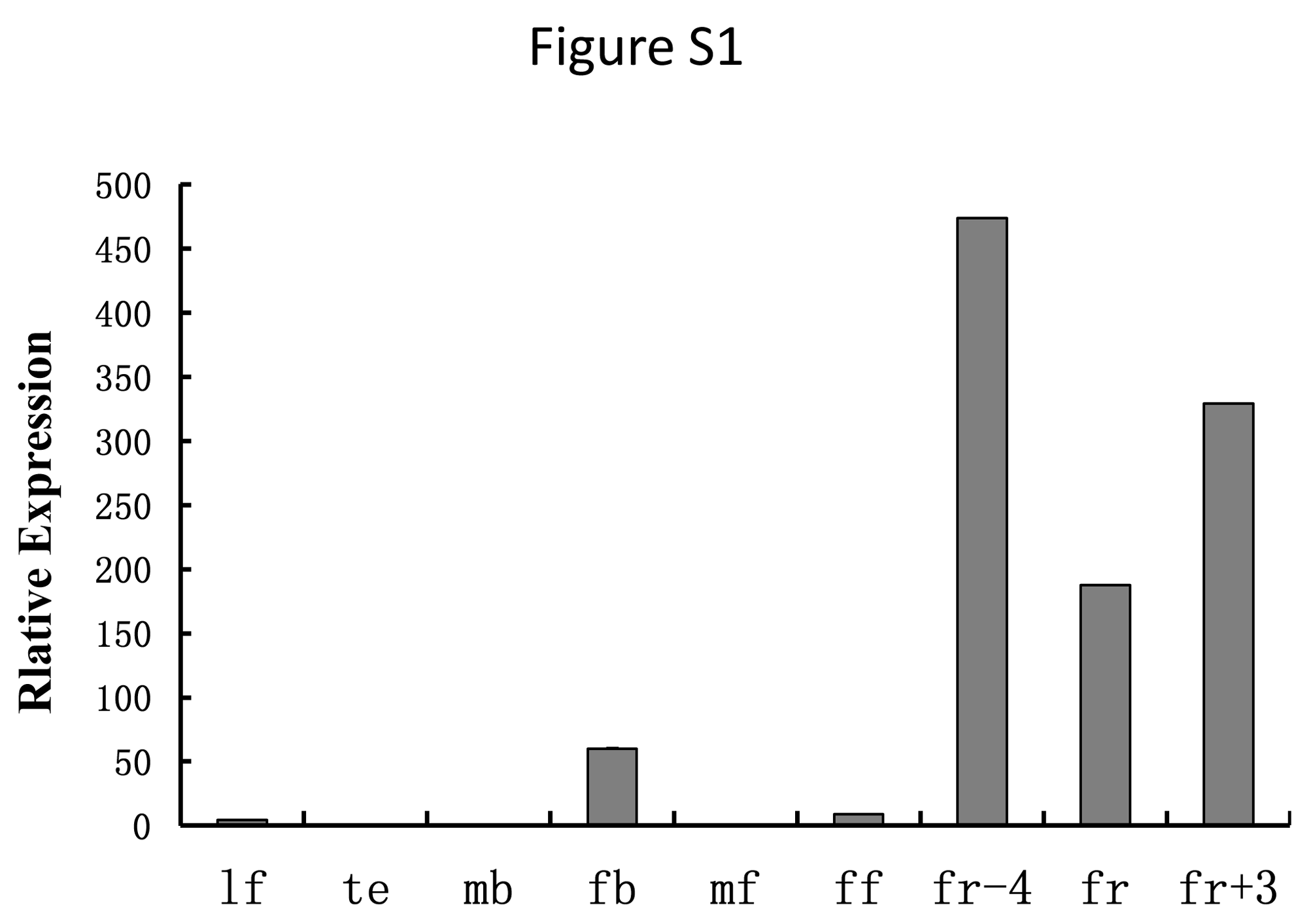

Supplement: Figure S1 — Quantitative RT-PCR (qRT-PCR) analysis of Cs001112 in different organs of cucumber. Csa001112 is predominately expressed in the fruit. lf: leaves, te: tendrils, mb: male flower buds, fb: female flower buds, mf: male flowers, ff: female flowers, fr-4: fruit of 4 days before flower opening, fr: fruit on flower opening, fr+3: fruit of 3 days after flower opening. Three biological replicates were used for each sample, and 18S rRNA was used as internal control. Bars represent the standard error. (TIF) [file pone.0086192.s001.tif]
