# Supplementary material for: Cucumber SUPERMAN Has Conserved Function in Stamen and Fruit Development and a Distinct Role in Floral Patterning
Source: PLoS One. 2014 Jan 23;9(1):e86192. doi: 10.1371/journal.pone.0086192 (PMC3900519; doi:10.1371/journal.pone.0086192)
Supplement: Table S1 — Oligonucleotide primers used in this study. (DOCX) [file pone.0086192.s002.docx]

**Table S1. Oligonucleotide primers used in this study**

| **Primers for cloning *CsSUP*** | |
| --- | --- |
| *CsSUP-F* | 5'-ATGAACACGTCGACGATTGGT-3' |
| *CsSUP-R* | 5'-CTAAGTTGAGTACCCTAATCGAAGC-3' |
| **Primers for RT-PCR** | |
| *q-CsSUP-F* | 5'-CATGGCCTCCAAGGAGTTAC-3' |
| *q-CsSUP-R* | 5'-CACCCTTCTTCCAATGACCT-3' |
| *q-Csa001112-F* | 5'-AGGGATAGGGCAAGAATGAGAC-3' |
| *q-Csa001112-R* | 5'-TGTAGTTCCAGATCAAGATCCTCA-3' |
| *Actin2-F* | 5'-CCTTCGTCTTGATCTTGCGG-3' |
| *Actin2-R* | 5'-AGCGATGGCTGGAACAGAAC-3' |
| *18S rRNA-F* | 5'-TCTGCCCGTTGCTCTGATG-3' |
| *18S rRNA-R* | 5'-TCACCCGTCACCACCATAG-3' |
| **Primers for *in situ* probes** | |
| *CsSUP-Sp6* | 5'-GATTTAGGTGACACTATAGAATGCTATGAACACGTCGACGATTGGT-3' |
| *CsSUP-T7* | 5'-TGTAATACGACTCACTATAGGGCTAAGTTGAGTACCCTAATCGAAGC-3' |
| **Primers for construction of 35S:*CsSUP* vector** | |
| *O-CsSUP-F* | 5'-GCTCTAGAGCATGAACACGTCGACGATTGGT-3' |
| *O-CsSUP-R* | 5'-TCCCCCGGGGGACTAAGTTGAGTACCCTAATCGAAGC-3' |
| **Primers for cloning *AtSUP* promoter** | |
| *pAtSUP-F* | 5'- AGAGGAGTGATTGAAAAGGGG-3' |
| *pAtSUP-R* | 5'- ATGTGCAAGCTCTTTCTTTTT-3' |
| **Primers for construction of *pAtSUP::CsSUP* vector** | |
| *pAtSUP–CsSUP-F* | 5'- CCATCGATGGAGAGGAGTGATTGAAAAGGGG-3' |
| *pAtSUP–CsSUP-R* | 5'- GCTCTAGAGCATGTGCAAGCTCTTTCTTTTT-3' |
